# Supplementary material for: LOX1- and PLP1-dependent transcriptional reprogramming is essential for injury-induced conidiophore development in a filamentous fungus
Source: Microbiol Spectr. 2023 Nov 9;11(6):e02607-23. doi: 10.1128/spectrum.02607-23 (PMC10714772; doi:10.1128/spectrum.02607-23)
Supplement: Supplemental figures and tables — The file contains 12 supplemental figures, three supplemental tables and expanded methods. [file spectrum.02607-23-s0008.pdf]

LOX1 and PLP1 dependent transcriptional reprogramming is essential for injury induced conidiophore development in a filamentous fungus

Martín O. Camargo-Escalante[1], Edgar Balcázar-López[1,2], Exsal M. Albores-Méndez[3], Robert Winkler[1], and Alfredo Herrera-Estrella[*1]

[1]Laboratorio Nacional de Genómica para la Biodiversidad-Unidad de Genómica Avanzada, Cinvestav. Km 9.6 Libramiento Norte Carretera Irapuato-León. 36824, Irapuato, Gto. México
[2]Present address: Departamento de Farmacobiología, Centro Universitario de Ciencias Exactas e Ingenierias, Universidad de Guadalajara, Guadalajara, Mexico
[3]Escuela Militar de Graduados de Sanidad, Universidad del Ejército y Fuerza Aérea Mexicanos, Secretaría de la Defensa Nacional, Mexico City, Mexico

* Corresponding author
Email: alfredo.herrera@cinvestav.mx
Phone: 52/462/1663000
Fax: (52)/462/6245849

Supplementary Information

This PDF includes:

- Materials and Methods
- Figures S1 to S13
- Tables S1 to S3
- Legends for Dataset S1 to S7
- References

1 Materials and Methods

1.1 Genomic DNA extraction and confirmation by Southern blot

Mycelium grown in Potato Dextrose Broth plates in complete darkness for 36h was harvested and immediately frozen using liquid nitrogen. For DNA extraction, a TENS-based method was used. Briefly, 300 mg of powdered mycelium in 500 μL (1 volume) of TENS (200 mM Tris-HCl (pH 8), 250 mM NaCl, 25 mM EDTA and 5% Sodium Dodecyl Sulfate (SDS)) was vortex during 5 min and then mixed with phenol: chloroform: isoamyl alcohol (25:24:1 v/v/v) 7 min in vortex. The aqueous phase recovered after centrifuging at 13458 g per 15 min was treated with 5 μL of RNAseA (incubation at 37°C per 30 min). Then, 1 volume of chloroform: isoamyl alcohol (24:1, v/v) was added to the aqueous phase and mixed by shaking in a vortex for 7 min; the aqueous phase was again recovered after centrifuging at 13458 g per 15 min (repeated twice). Next, nucleic acids were precipitated, adding 0.8 volume of isopropanol to the aqueous phase mixing gentlemanly, and then the DNA pellet was recovered by centrifuging 13458 g per 10 min. Later, the DNA pellet was cleaned twice with 1 mL 70 % EtOH and dissolved in 50 μL sterile, deionized water. For confirmation by Southern blot, 10 μg genomic DNA of each candidate mutant and WT were digested with *Pst*I and *Sac*I for Δ*plp1* and *Hinc*II and *Bsa*I for Δ*lox1*. After digestion, DNA was separated by electrophoresis in a 1% agarose gel and transferred onto a Hybond-N+ membrane (Amersham) using 2X and 20X SSC (3.0 M NaCl, 0.3 M sodium citrate) solution. The details of the DNA transfer in this work were followed as previously described [3]. The probes (E-2) (Fig. S2 diagram) were labeled with [α-^{32}P] dCTP by random priming using the Megaprime DNA labeling System, dCTP (Amersham). Hybridization was carried out in an oven at 60°C overnight and revealed in X-ray films (BioMax MR, KODAK).

1.2 RNA extraction and quantitative-PCR (qPCR)

Mycelium grown on a cellophane sheet placed over PDA medium plates was harvested and immediately frozen using liquid nitrogen. The TRIZOL method was used for RNA extraction: 1 mL of TRIZOL was added to 500 mg of powdered mycelium collected in 1.5 mL Eppendorf, mixed by shaking in a vortex for 10 min and incubated for 3 min at room temperature. Then, 200 μL of chloroform: isoamyl alcohol (24:1, v/v) was added to the extraction tube and mixed in a vortex for 3 min. The aqueous phase was recovered after centrifuging at 13458 g (4°C) per 15 min, and then 200 μL of chloroform was added (mixed in a vortex for 3 min). The aqueous phase, recovered again (1 volume = recovered volume) by centrifuging (13458 g/15 min at 4°C), was treated with two volumes of 100% EtOH and 1/10 3M sodium acetate (incubated at -20°C for 20 min). Then, the pellet was collected by spinning at 6021 g/10 min at 4°C and dissolved in 300 μL RNAse-free deionized water. Next, RNA was precipitated by adding 100 μL of 2 M LiCl (incubate for 2 h in ice). The RNA pellet was collected by centrifuging and cleaned first with 1 mL 70 % EtOH (RNAse-free solution) and then with 1 mL 75 % EtOH (RNAse-free solution). Finally, the RNA pellet was dissolved in 50 μL RNAse-free deionized water. For gene expression evaluation, we designed primers for qPCR (Table S1) to produce amplicons around 150 pb. cDNA was synthesized using SuperScript III Reverse Transcriptase (Invitrogen) with 9 μg DNase I-treated RNA. qPCR was performed with 1.5 μL diluted cDNA (diluted 1:10) as templated, 10 μL SYBR Green PCR Master Mix (Applied Biosystems), 0.25 μM of final concentration for each primer and water up to 20 μL total volume. The qPCR was carried out in an Applied Biosystems 7500 Real-Time PCR System (Thermo Fisher) as follows: 94°C for 10 min, 40 cycles of 94°C for 30 s, 60°C for 30 s, and 72°C for 40 s. Each product's melt curve, from 60°C to 95°C at an increment of 0.2 °C/s, produced a single melting point. All qPCR reaction was repeated four times.

2 Supplementary Figures

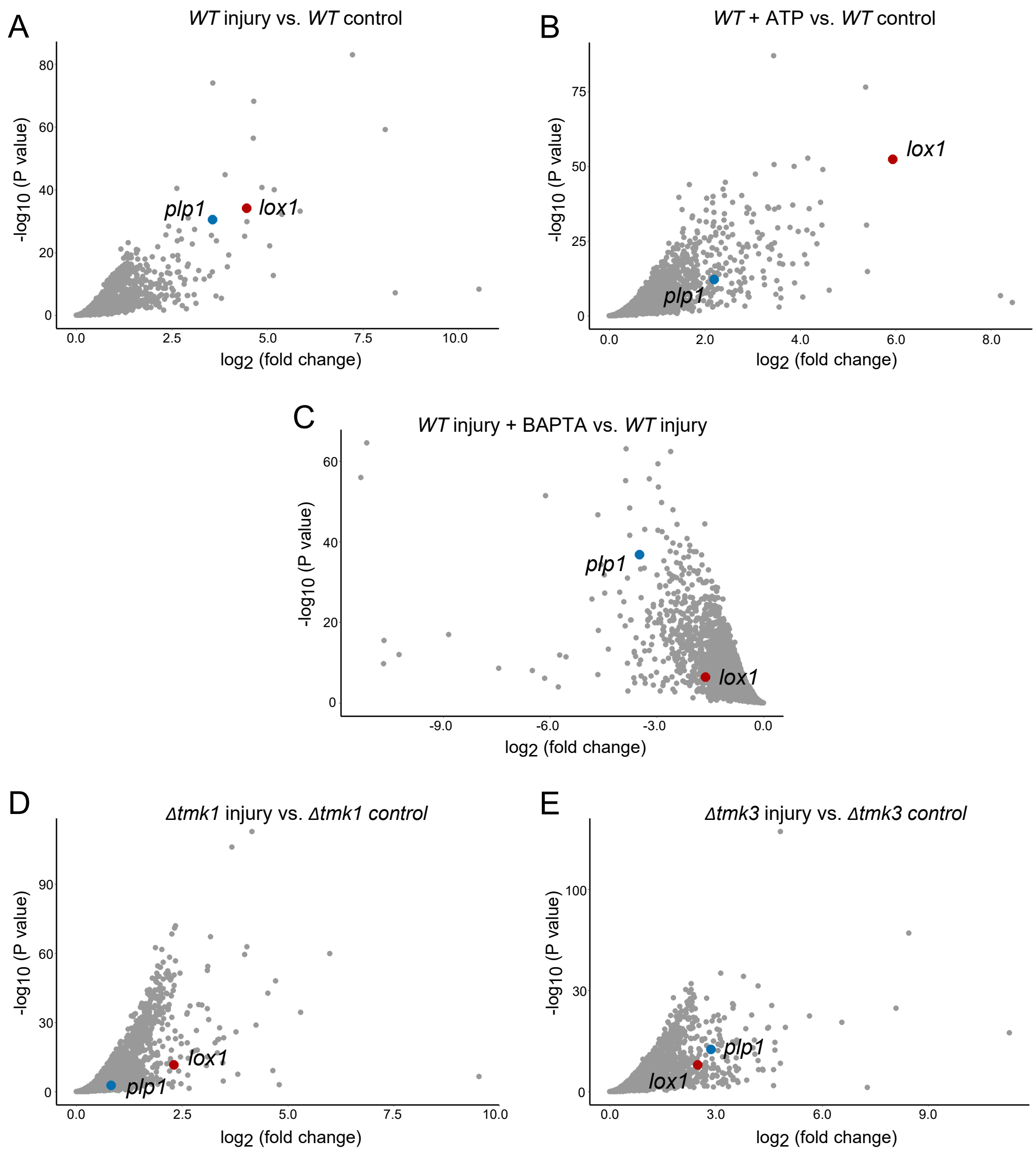

Supplementary Figure 1. Damage signaling by eATP and Ca^{2+} induces the *lox1* and *plp1* genes to produce 13-HODE. Volcano plot of -$\log_{10}$(P value) vs $\log_2$(Fold change) of differentially expressed *lox1* and *plp1* (FDR < 0.05) in the context of mechanical damage. To analyze and visualize the transcriptional behavior of *lox1* and *plp1*, we used the differential expression data of damage perception and signaling mechanisms previously reported [2]. The data are available in the Gene Expression Omnibus database (accession number: GSE115811).

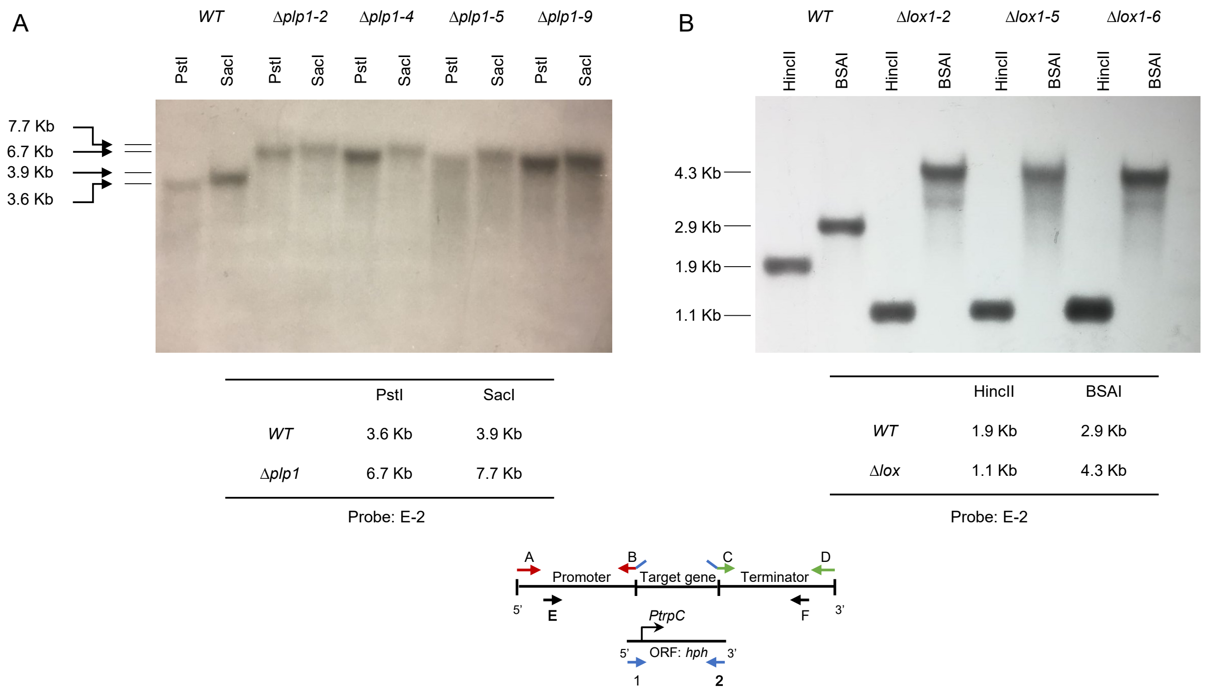

Supplementary Figure 2. Mutant confirmation by Southern blot. DNA of each candidate mutant was digested *PstI* and *SacI* for Δ*plp1* (A) and *HincII* and *BsaI* for Δ*lox1* (B). A probe (E-2) was used for hybridization, according to the construction for homologous recombination generated by double-joint PCR.

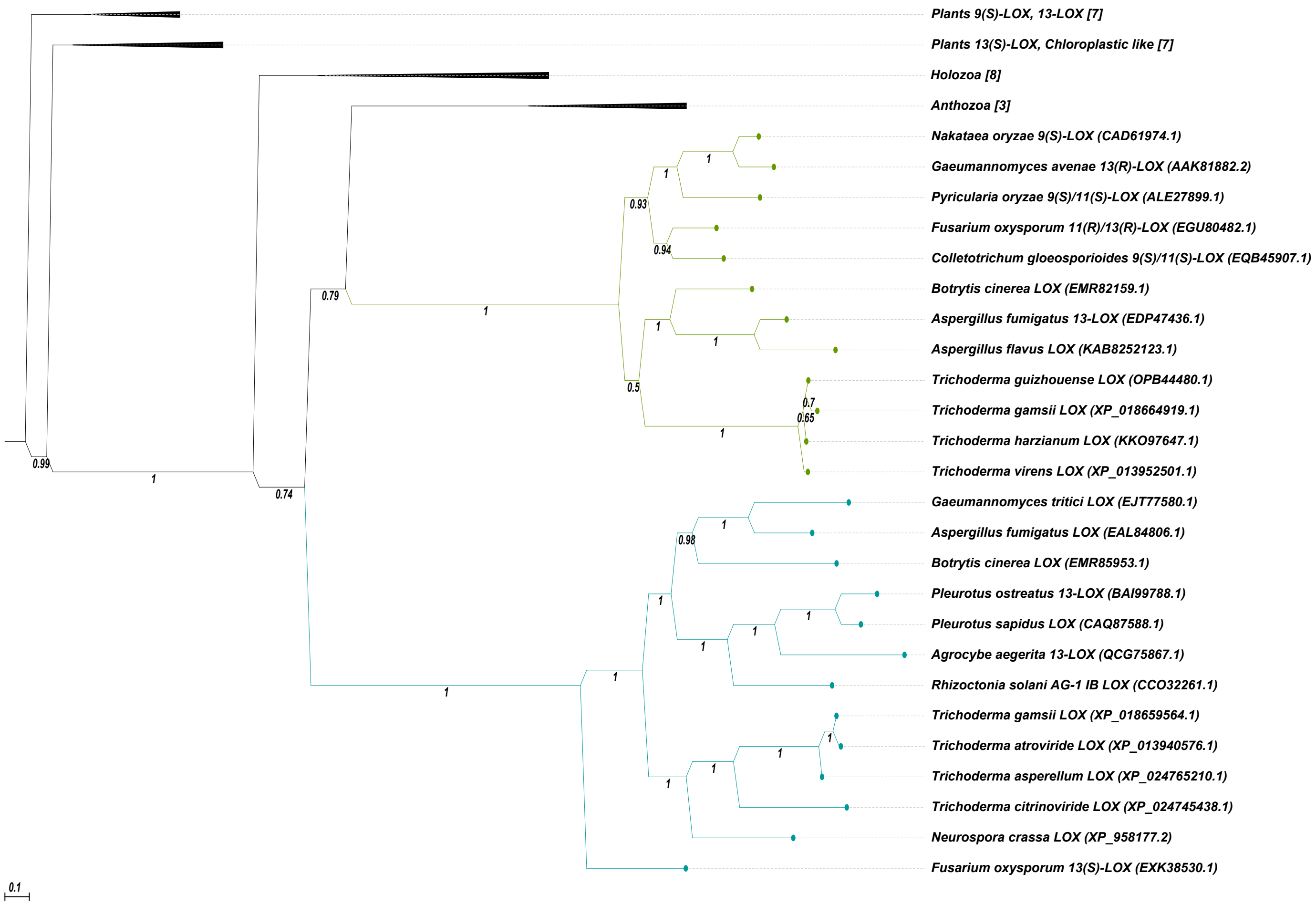

Supplementary Figure 3. Phylogenetic tree of lipoxygenases in fungi, animals, and plants. Bayesian phylogram based on the analysis of 55 orthologous proteins in plants, animals, and fungi (MCMC, 1 million generations). Blue branches: Fe-dependent lipoxygenases and green branches: Mn-dependent lipoxygenases. Scale bars represent the average number of amino acid substitutions per site. Numbers below each branch represent the percentage of the posterior probability. The phylogenetic tree shows that fungal lipoxygenases are closer to animal (Holozoa) than to plant lipoxygenases.

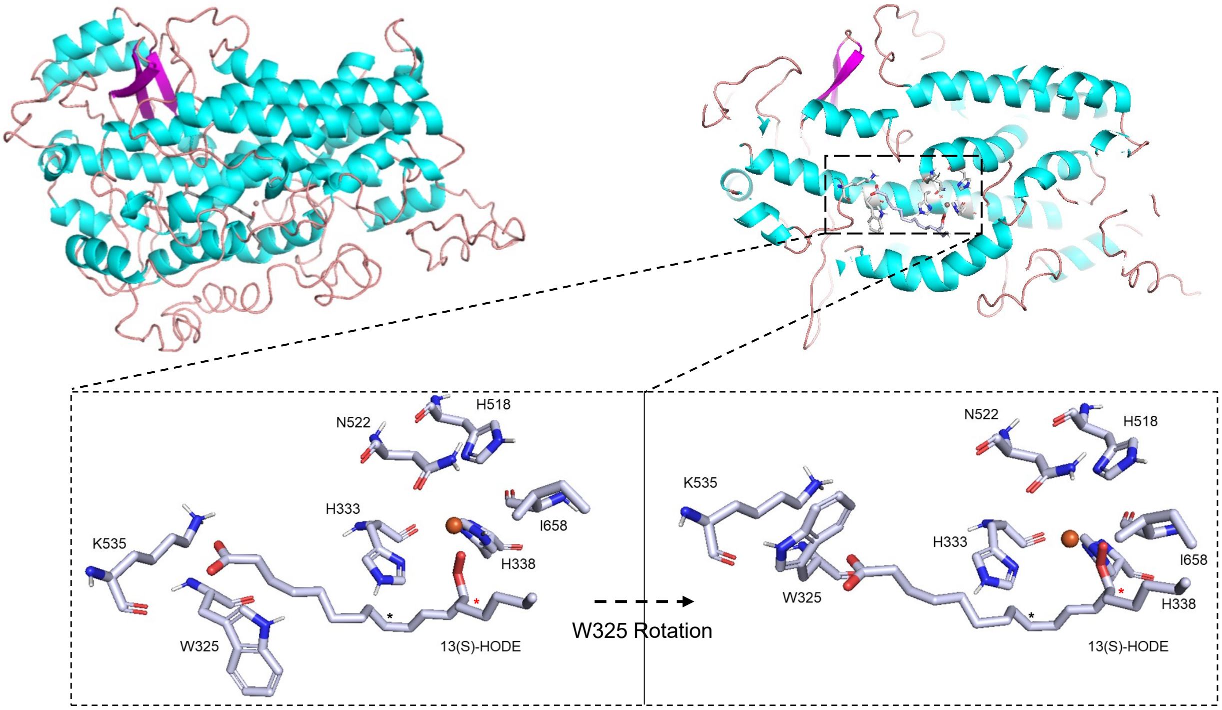

Supplementary Figure 4. ***In silico*** **modeling of** ***Trichoderma atroviride*** **lipoxygenase.** Crystallized *Fusarium graminearum* lipoxygenase (PDB: 6NS2_A) was used as a template for structure prediction by I-TASSER. *T. atroviride* LOX protein conserves residues involved in coordinating the metallic cofactor (Fe): His-333, His-338, Asn-522, His-518, and Ile-658, and dispensable amino acids for the regio- and stereo-specific di-oxygenation reaction observed in animal and plant LOX: K535 and W325. The last two localized at the bottom very close to the predicted ligand (13-HODE, C-score = 0.33). If Trp-325 were rotated, it could work like a shell to block a salt bridge formation between Lys-535 and the carboxylic group of the fatty acid and avoid deeper penetration, thus favoring oxidation in C-13 [1]. Parameters of the model predicted by I-TASSER: C-score = 0.55; estimated TM-score = 0.79±0.09; estimated RMSD = 6.7±4.0 Å. BLASTp: 6NS2_A as subject: Query Cover (QC) = 98%, Percent Identity (PI) = 38.9%. Asterisks indicate positions C-9 (black) and C-13 (red) of linoleic acid.

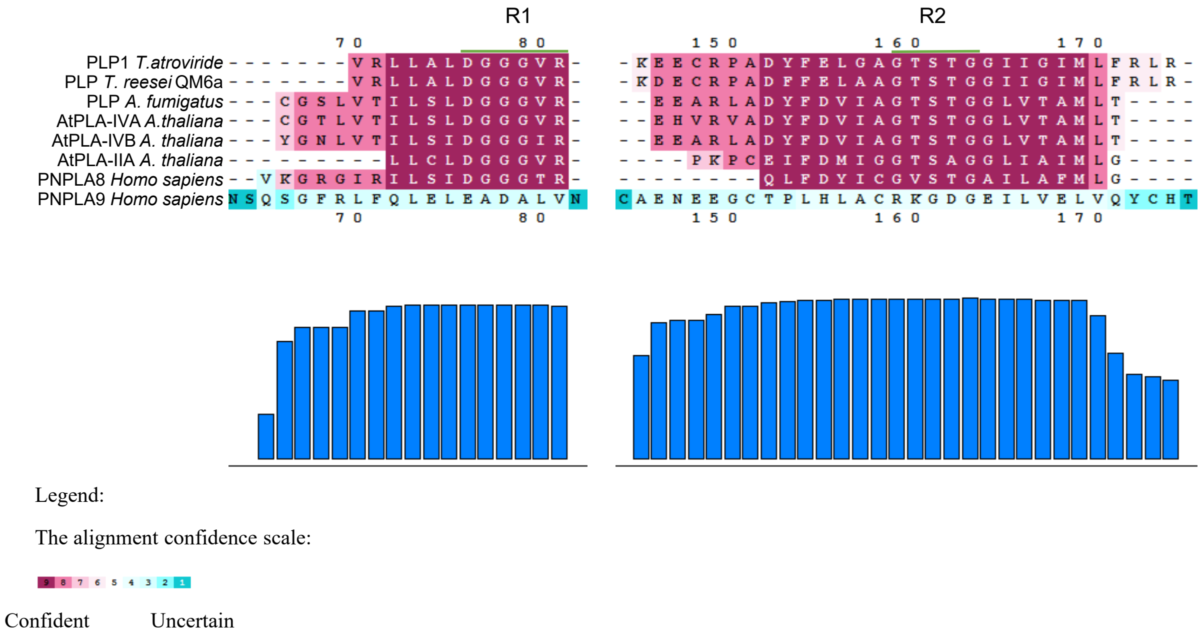

Supplementary Figure 5. Alignment analysis of patatin-like phospholipase amino acid sequences homologs in fungi, animals, and plants. Region 1 (R1) represents the phosphate binding motif; Region 2 (R2) represents the catalytic motif. Both motifs are conserved in some patatin-like phospholipases in fungi, animals, and plants. At the amino acid level, PLP1 has three close acyl hydrolase orthologs (PSI-Blast and DELTA-Blast) in *A. thaliana*, AtPLA-IVA (QC = 91%; PI = 20.36%), AtPLA-IVB (QC = 83%; PI = 21.82%) and AtPLA-IIA (QC = 86%, PI = 19.70%); and an in humans, PNPLA8 (QC = 82%, PI = 22.43%). Patatin-like phospholipase amino acid sequences: *T. atroviride* PLP1 (XP_013938719.1), *T. reesei* QM6a PLP (XP_006964372.1 hp), *A. fumigatus* PLP (KAH1492946.1 hp), *A. thaliana* AtPLA-IVA (NP_568015.1), *A. thaliana* AtPLA-IVB (NP_195423.1), *A. thaliana* AtPLA-IIA (NP_180224.1), *Homo sapiens* PNPLA8 (AAD08847.1), *Homo sapiens* PNPLA9 (AAC97486.1).

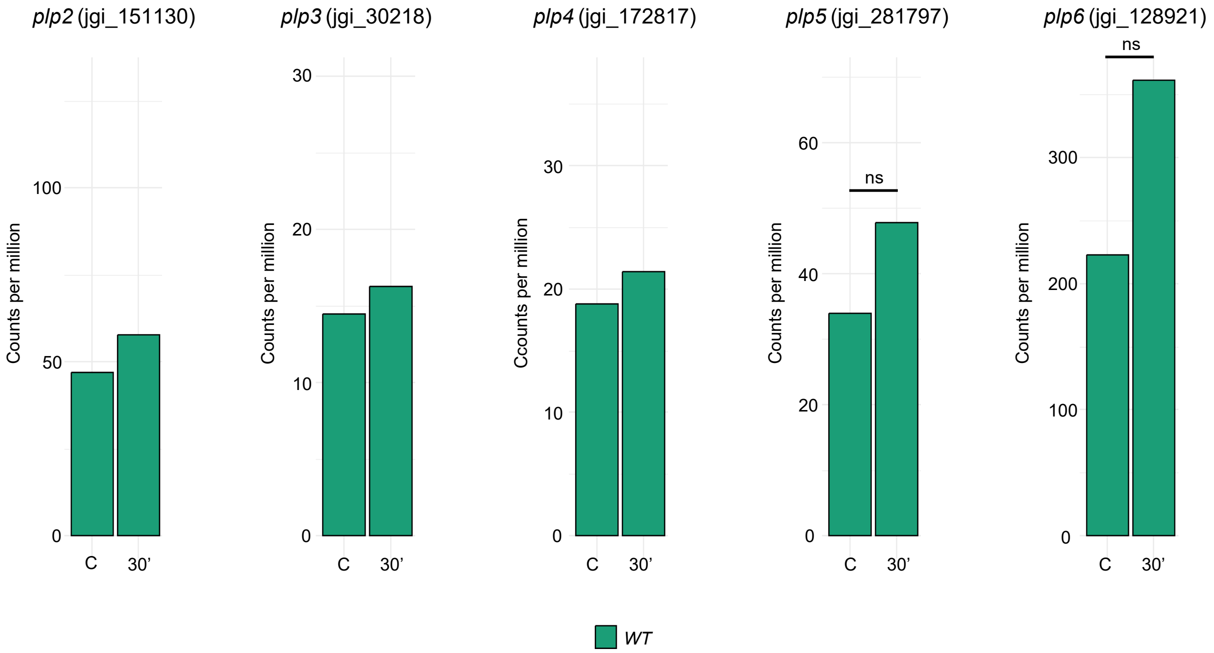

Supplementary Figure 6. Five phospholipases with a patatin-like domain homolog to *plp1* expressed at basal level in response to mechanical damage. Counts per million of patatin-like phospholipase in the control condition and 30 min after injury in the WT strain [2]. The data were taken from Gene Expression Omnibus with Serie accession number GSE115811: C, undamaged control; 30', 30 min after injury; ns, not significant.

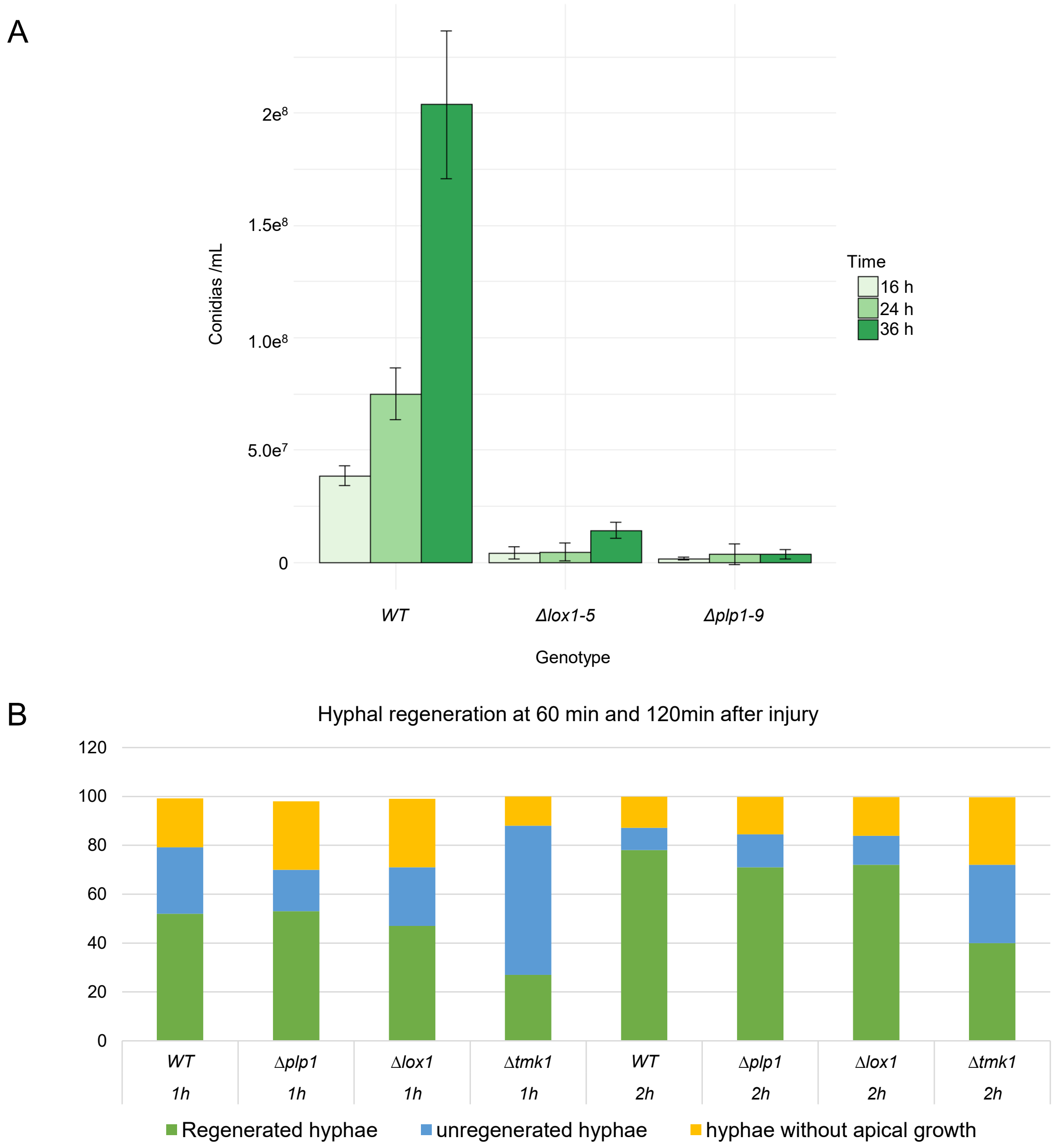

Supplementary Figure 7. Injury-induced conidiation quantification and hyphal regeneration analysis at 60' and 120' after injury. (A) Quantification of conidia produced after injury using other independently transformed *lox1* and *plp1* mutants, Δ*lox1*-5 and Δ*plp1*-9. (B) The proportion of regenerated and unregenerated hyphae. The number of regenerated hyphae increases with time in the mutant and WT strains, except in Δ*tmk1*, where a slight increase was observed.

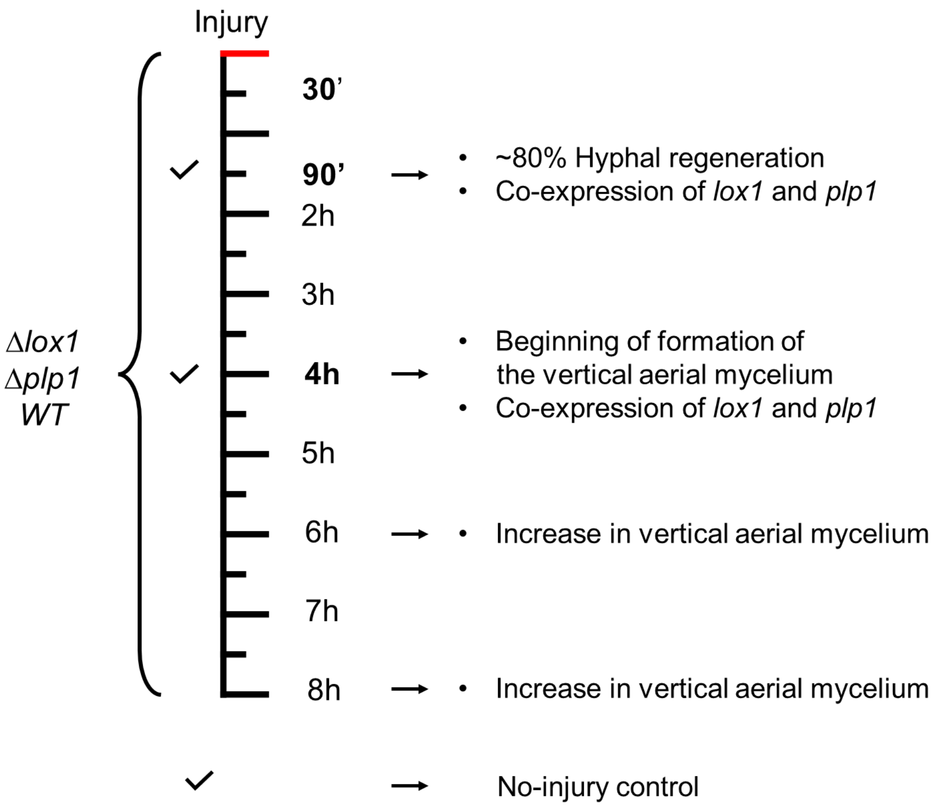

Supplementary Figure 8. Experimental strategy to find genes related to injury-induced conidiation. We based our experimental design on finding genes and biological processes that *lox1* and *plp1* could regulate after the regeneration window. We collected mycelium at 90 min, early response, and 4 h after injury before aerial mycelium appeared. The ticks indicate the times in which the mycelium was collected.

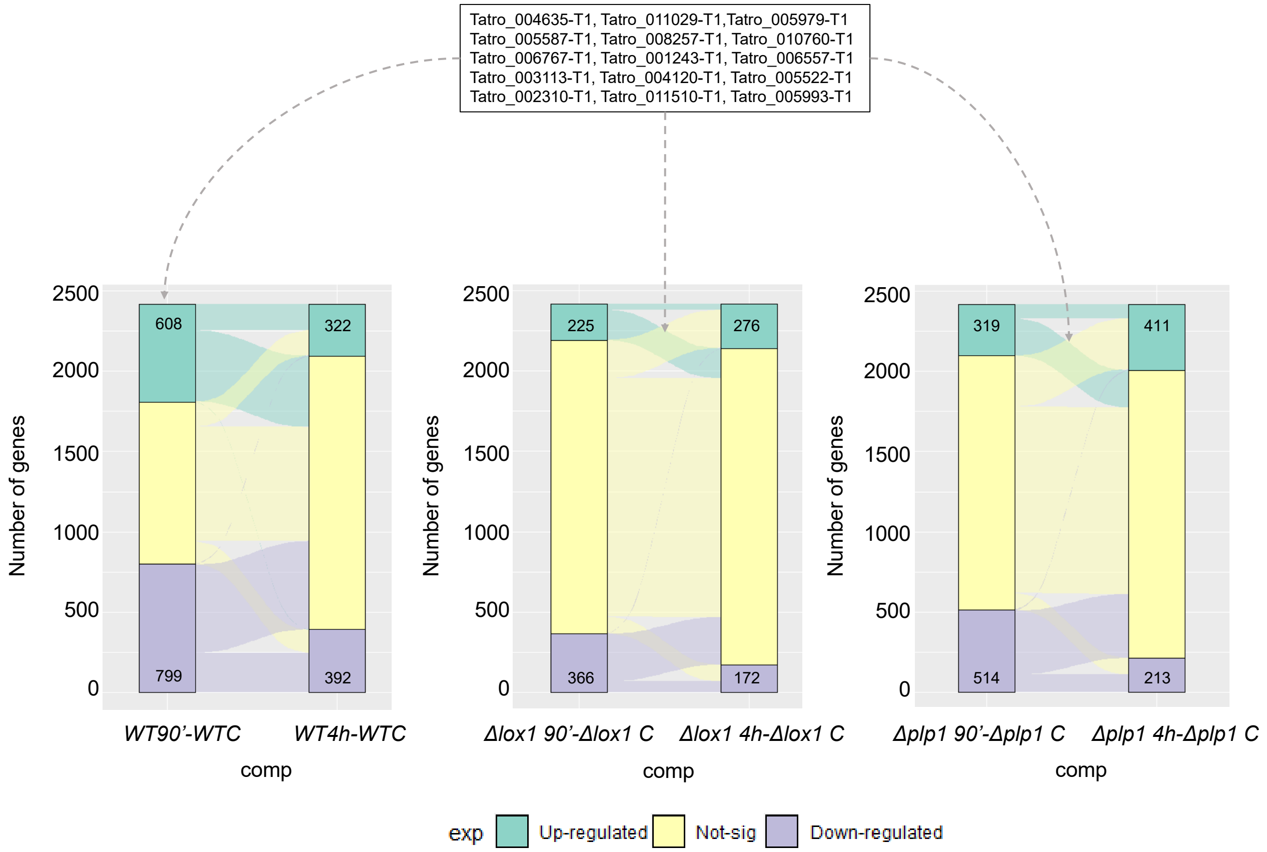

Supplementary Figure 9. Time course behavior of genes up-regulated or down-regulated in damage response. Plots represent the matrix of differentially expressed genes in response to wounding in WT and mutants of *lox1* and *plp1*. The colored boxes indicate each contrast's up-regulated, down-regulated, and non-significant genes (comp). The mutation in *lox1* and *plp1* affects the timing of appropriate expression of some genes (black line box) up-regulated in response to wounding in WT (WT 90'–WT C). comp = comparison (contrast); exp = indicates the categories up-regulated, down-regulated, or not significant.

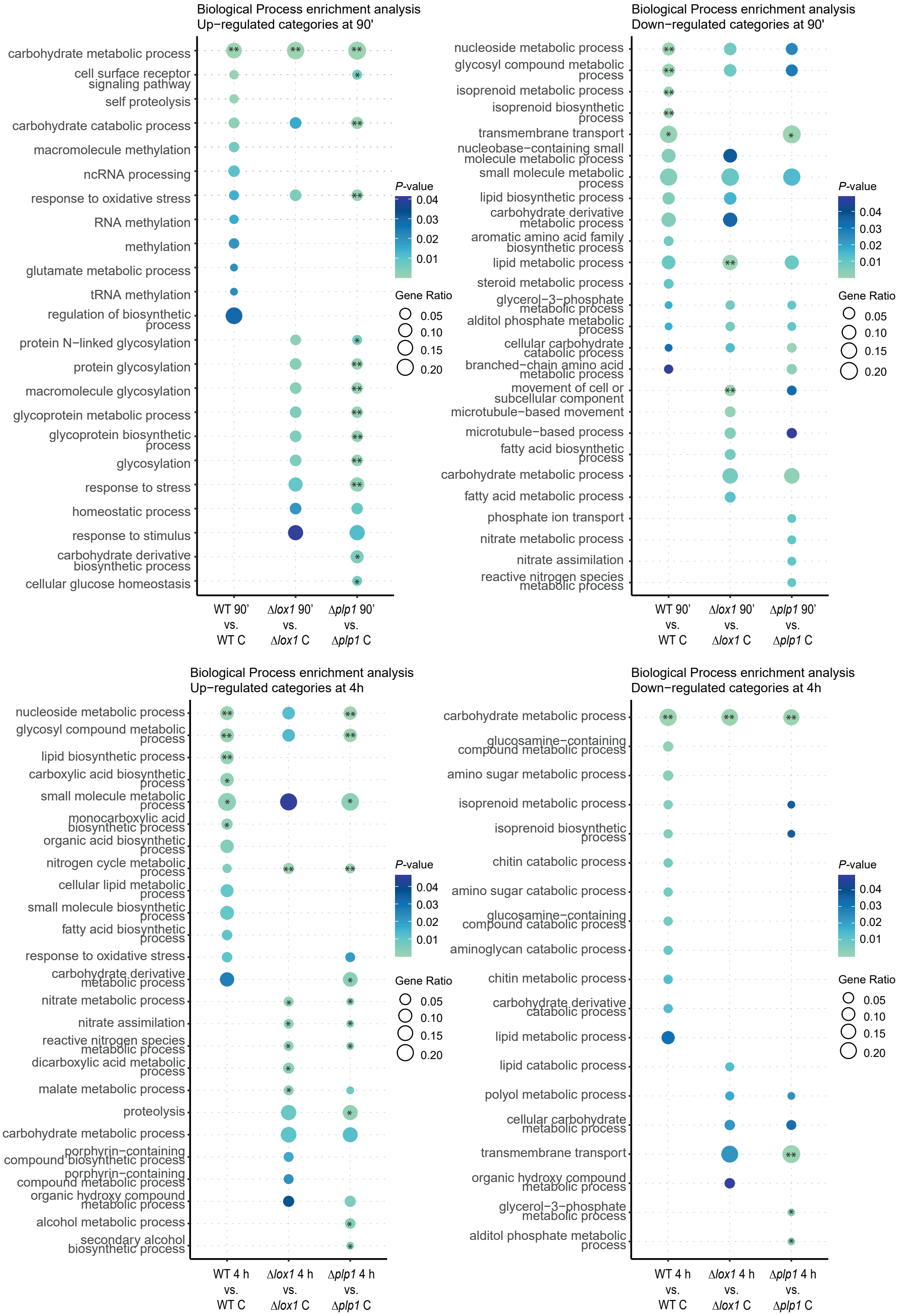

Supplementary Figure 10. Representation of the enriched functional categories of Biological Processes GO terms in the damage transcriptional response in mutant and WT strains. The color of the circles represents the *P*-value scale ($P < 0.05$), and size indicates the Gene Ratio (ratio or percentage of total differential expressed genes associated with a GO term) in each biological process. Asterisks denote the FDR adjusted *P*-value: *, *P* adjust < 0.1; **, *P* adjust < 0.05. No asterisk indicates raw *P*-value < 0.05.

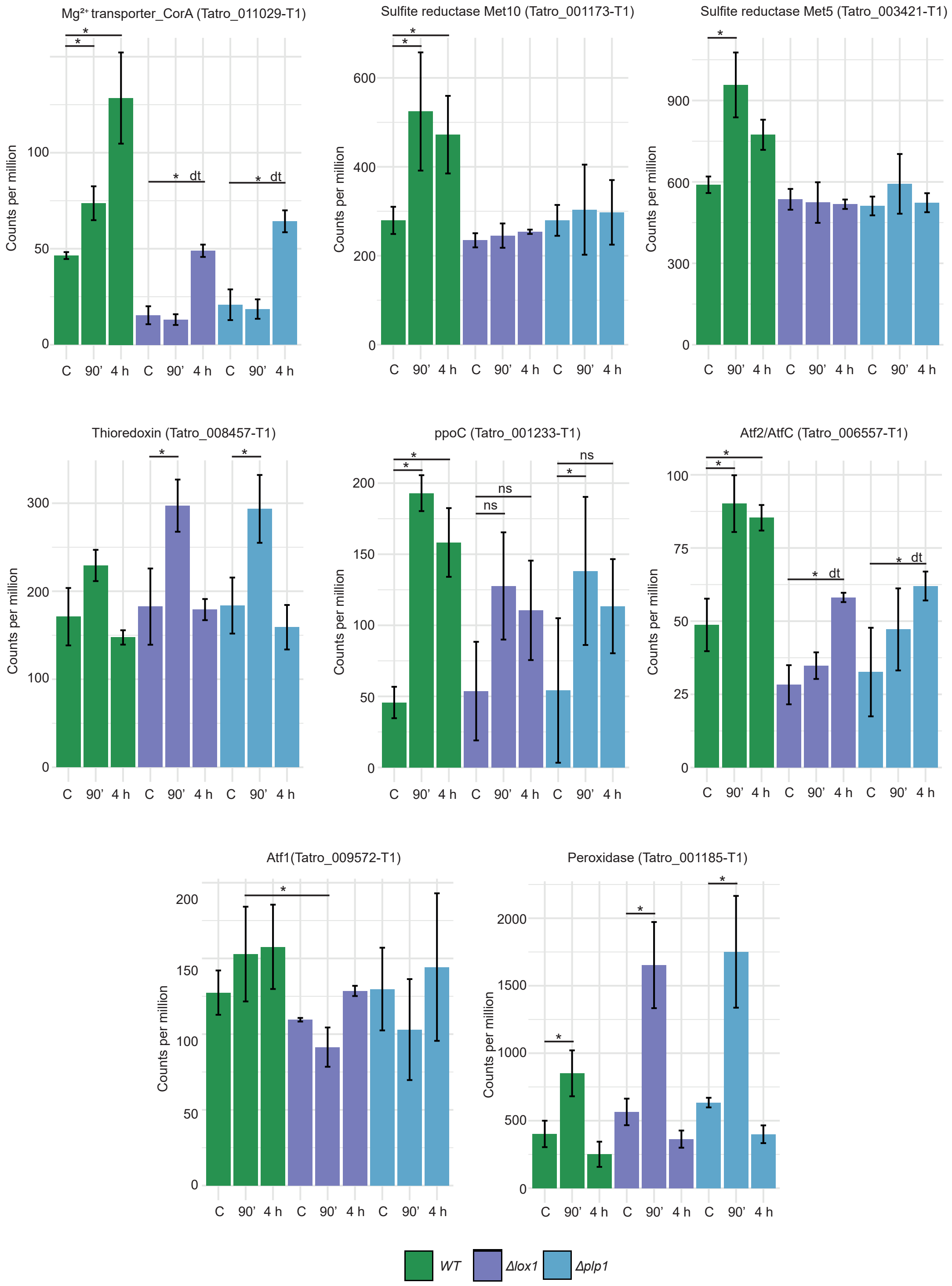

Supplementary Figure 11. Comparison of the expression levels of diverse genes in the mutant and WT strains related to TFs, signaling, and metabolism. Bar plot of counts per million (CPM) of some genes related to TFs, signaling, and metabolism. Black lines represent the pairwise comparison; the asterisk on the black line indicates a statistically significant contrast (*P*-value < 0.05; FDR < 0.05); ns, not statistically significant. Also, the mutation affects the timing of appropriate expression (dt) of Tatro_011029-T1 and Tatro_006557-T1 (Fig. 6B and C; Fig. S107), leading to late up-regulation.

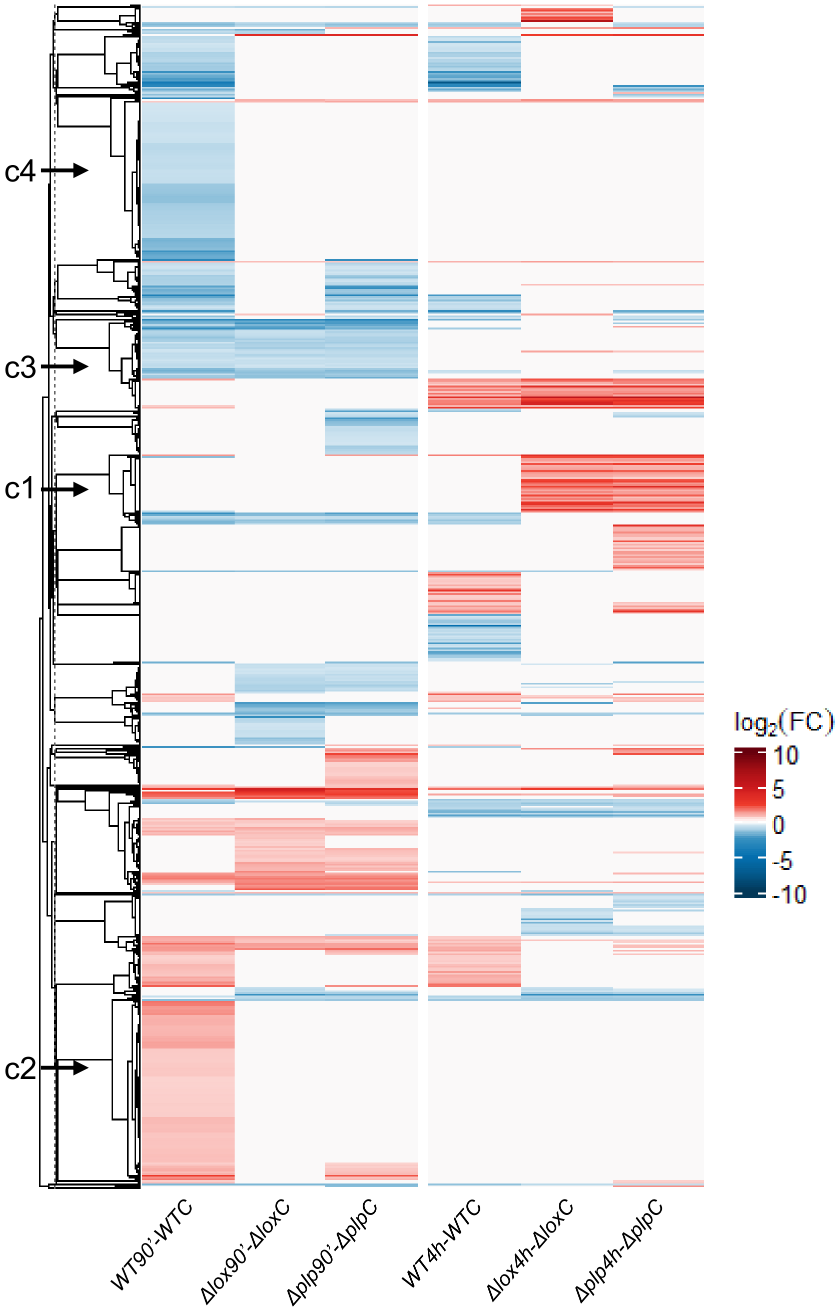

Supplementary Figure 12. Heatmap of fold change of up-regulated and down-regulated genes in wound response in mutant and WT strains. The hierarchical clustering of rows was determined using a Pearson correlation-based distance measure. The hierarchical clustering was done using the *eclust* package and the "average" method, generating 42 groups. The up-regulated and down-regulated genes ($\log_2$FC > |0.559|) in wound response in WT and mutants (Δ*lox1* and Δ*plp1*) resulted from a Bayes quasi-likelihood F-test to a specified threshold, LFC = 0.

3 Supplementary Tables

Supplementary Table 1. Primers used in this work.

Gene name	ID (JGI)	Label name	Sequence	Use
Lipoxygenase	33350	LipR1Fwd	GACCACGTACCCCAAGAGATCC	Double-joint PCR
Lipoxygenase	33350	LipR1Rev	CCT TCA ATA TCA GTT AAC GTC GAT CCG TCA CAG CCG CAC AAG CCC AGT TG	Double-joint PCR
Lipoxygenase	33350	LipR2Fwd	CAC TCG TCC GAG GGC AAA GGA ATA GCT CTC CCC ATT GGT CGC GAG	Double-joint PCR
Lipoxygenase	33350	LipR2Rev	CTTATGTATTCCGGTATCCTCTCACG	Double-joint PCR
Lipoxygenase	33350	NestLip1	GTTGGCAGCGTCCTGGGCAG	Double-joint PCR
Lipoxygenase	33350	NestLip2	GATCAAATCCGCCTTGGTACGCCG	Double-joint PCR
Phospholipase	28577	PhosR1Fwd	CAA CCT GGC TAG ACC TGG GAC	Double-joint PCR
Phospholipase	28577	PhosR1Rev	CCT TCA ATA TCA GTT AAC GTC GAT C GGA CAA TCT GGG TGG CAA GAG TC	Double-joint PCR
Phospholipase	28577	PhosR2Fwd	CAC TCG TCC GAG GGC AAA GGA ATA GGA G GAG CTG AAG ACG TTG GCG	Double-joint PCR
Phospholipase	28577	PhosR2Rev	ATG CTC CAA GAT CGC GTG CCC	Double-joint PCR
Phospholipase	28577	NestPhos1	GAG CGT GAC AGT GCG TTC ATG G	Double-joint PCR
Phospholipase	28577	NestPhos2	CTG CCG AAG GGT GTA TCG TAG G	Double-joint PCR
Hygromycin B phosphotransferase		HygORFfw	GAAAAGTTCGACAGCGTCTCCG	hph integration verification
Hygromycin B phosphotransferase		HygORFrv	CTTTGCCCTCGGACGAGTGCT	hph integration verification
Hygromycin B phosphotransferase		HPH-FW	GACAGAAGATGATATTGAAGGAGC	Double-joint PCR
Hygromycin B phosphotransferase		HPH-RV	GATTTCAGTAACGTTAAGTGGAT	Double-joint PCR
Lipoxygenase	33350	QpcrF1-33350	GCAGAGATATCCAACGACACTC	qRT-PCR
Lipoxygenase	33350	QpcrR1-33350	GGC TTG GTA GAA GTT TTG CAG	qRT-PCR
Patatin-like phospholipase/iPLA2	28577	QpcrF1-28577	AAGTTGACATTCTGGGACGG	qRT-PCR
Patatin-like phospholipase/iPLA2	28577	QpcrR1-28577	AAG CTG GAA CCA TGA CTC TG	qRT-PCR

Supplementary Table 2. Characteristic of processed libraries. The table displays the barcode number, quantity of gene models, genes with zero reads and alignment statistic for each library.

ID	Gene models	Zero %	Aligned read quantity	Library label	Genotype
L02_74	12024	14.06%	6625839	lx4h-1	Δ*lox1*
L02_83	12024	15.00%	6509454	lx4h-2	Δ*lox1*
L02_91^{tb}	12024	17.01%	4066732	lx4h-3^{sl}	Δ*lox1*
L02_92^{tb}	12024	17.27%	3894554	lx4h-3^{sl}	Δ*lox1*
L02_73	12024	16.04%	5046895	lx90m-1	Δ*lox1*
L02_82	12024	13.64%	7995011	lx90m-2	Δ*lox1*
L02_89^{tb}	12024	19.89%	2543217	lx90m-3^{sl}	Δ*lox1*
L02_90^{tb}	12024	19.59%	2687534	lx90m-3^{sl}	Δ*lox1*
L02_72	12024	14.55%	6214866	lxc-1	Δ*lox1*
L02_81*	12024	80.24%	5422	lxc-2	Δ*lox1*
L02_88	12024	17.26%	4264273	lxc-3	Δ*lox1*
L02_77	12024	14.37%	6488568	pp4h-1	Δ*plp1*
L02_44	12024	14.59%	5587826	pp4h-2	Δ*plp1*
L02_96	12024	17.51%	5033418	pp4h-3	Δ*plp1*
L02_76	12024	16.76%	5511116	pp90m-1	Δ*plp1*
L02_43	12024	12.80%	9496850	pp90m-2	Δ*plp1*
L02_95	12024	14.60%	7048216	pp90m-3	Δ*plp1*
L02_75	12024	15.47%	5805898	ppc-1	Δ*plp1*
L02_84	12024	13.83%	7289807	ppc-2	Δ*plp1*
L02_93^{tb}	12024	18.74%	2549776	ppc-3^{sl}	Δ*plp1*
L02_94^{tb}	12024	15.64%	5200691	ppc-3^{sl}	Δ*plp1*
L02_71	12024	15.71%	5675170	wt4h-1	WT
L02_80	12024	13.30%	8538438	wt4h-2	WT
L02_87	12024	14.78%	5917107	wt4h-3	WT
L02_70	12024	16.49%	4662020	wt90m-1	WT
L02_79	12024	16.32%	5091339	wt90m-2	WT
L02_86	12024	16.78%	3609486	wt90m-3	WT
L02_69	12024	15.14%	4315904	wtc-1	WT
L02_78	12024	12.26%	8471696	wtc-2	WT
L02_85	12024	13.60%	7359173	wtc-3	WT

* Indicates discarded library, ^{tb} indicates the same library with two different barcodes (^{sl}).

The number of ID indicates barcode number and its corresponding label (Library label).

Supplementary Table 3. Phospholipases of *T. atroviride* with a patatin-like domain homolog to patatin-like phospholipase 1.

ID Query	Organism	Query Cover	E-value	Identity (%)	Accesion length
151130	*T. atroviride*	94%	7e-49	18.83%	626
30218	*T. atroviride*	72%	8e-16	14.63%	762
172817	*T. atroviride*	62%	3e-15	16.94%	775
281797	*T. atroviride*	57%	8e-09	16.30%	564

DELTA-BLASTp analysis using as query the amino acid sequence of *plp1* (jgi_28577).

4 Supplementary Datasets

Supplementary Data Set 1. Differentially expressed genes (DEGs) and their annotation in the comparison WT injury–WT control, Δ*lox1* injury–Δ*lox1* control, and Δ*plp1* injury–Δ*plp1* control. The spreadsheet includes lists of DEGs and differential expression statistical support in each comparison and analyzed stage (control, 90', and 4h after injury), and functional and protein domain annotation (Pfam).

Supplementary Data Set 2. Enrichment analysis of Biological Process GO terms shared between Δ*lox1* injury–Δ*lox1* control and Δ*plp1* injury–Δ*plp1* control. List of up-regulated or down-regulated BP GO terms shared in both mutants in the response to injury.

Supplementary Data Set 3. Enrichment analysis of Biological Process GO terms in the contrasts WT injury–WT control, Δ*lox1* injury–Δ*lox1* control, and Δ*plp1* injury–Δ*plp1* control. List of up-regulated or down-regulated BP GO terms in each comparison and stage analyzed (control, 90', and 4h after injury) and statistical support of the enrichment analysis.

Supplementary Data Set 4. Enrichment analysis of KEGG pathways in the contrasts WT injury–WT control, Δ*lox1* injury–Δ*lox1* control, and Δ*plp1* injury–Δ*plp1* control. The spreadsheet includes up- and down-regulated KEGG pathways and their statistical support in each analyzed contrast and condition (control, 90', and 4h after injury).

Supplementary Data Set 5. Enrichment analysis of Biological Process GO terms of WT-unique genes (WUGs). The lists include up-regulated and down-regulated BP GO terms and their statistical support of induced or repressed WUGs (WT injury–WT control) at 90' and 4h after injury.

Supplementary Data Set 6. List of up-regulated WT-unique genes (WT injury–WT control). The spreadsheet includes a list of up-regulated genes at 90' and 4h after injury, differential expression statistical support, and functional and protein domain annotation (Pfam).

Supplementary Data Set 7. Differentially expressed genes (DEGs) in the comparison Δ*lox1*–WT and Δ*plp1*–WT in each condition. The spreadsheet includes a list of DEGs and statistical support in each contrast and stage (control, 90', and 4h after injury), and functional and protein domain annotation (Pfam).

References

[1] F. Brodhun, A. Cristobal-Sarramian, S. Zabel, J. Newie, M. Hamberg, and I. Feussner. An Iron 13S-Lipoxygenase with an α-Linolenic Acid Specific Hydroperoxidase Activity from Fusarium oxysporum. *PLoS ONE*, 8(5), 2013.

[2] E. Medina-Castellanos, M. Villalobos-Escobedo, M. Riquelme, N. D. Read, C. Abreu-Goodger, and A. Herrera-Estrella. Danger signals activate a putative innate immune system during regeneration in a filamentous fungus. *PLoS Genetics*, 14(11):e1007390, 2018.

[3] E. Southern. Southern blotting. *Nature Protocols*, 1(2):518–525, 2006.
